# Professional Autonomy in Nursing Practice: A Qualitative Study From the Perspective of Nurse Managers

**DOI:** 10.1155/jonm/4380993

**Published:** 2026-07-21

**Authors:** Gözde Özaras Öz, Rümeysa Yildiz

**Affiliations:** ^1^ Department of Nursing Fundamentals and Management, Faculty of Health Sciences, Çankırı Karatekin University, Cankiri, Turkey, karatekin.edu.tr; ^2^ Department of Nursing Fundamentals, Faculty of Health Sciences, Çankırı Karatekin University, Cankiri, Turkey, karatekin.edu.tr

**Keywords:** interprofessional relations, leadership, nurse manager, organizational culture, professional autonomy, qualitative research

## Abstract

**Aim:**

The aim of this study was to examine nurse managers’ perceptions of nurses’ professional autonomy, how professional autonomy is enacted and supported in clinical care settings, and the managerial and organizational processes influencing autonomous nursing practice.

**Design:**

A qualitative descriptive study.

**Background:**

Professional autonomy is a core component of nursing practice and is associated with care quality. While previous studies have predominantly examined autonomy from nurses’ perspectives, evidence remains limited regarding nurse managers’ views and their role in shaping organizational conditions that enable or constrain autonomous nursing practice.

**Methods:**

This study was conducted in a 300‐bed public hospital in Türkiye. Fifteen nurse managers working in acute care units participated in semistructured interviews. Data were analyzed using inductive qualitative content analysis and reported in accordance with the COREQ guidelines.

**Results:**

Three main categories emerged: (1) integration of nurses’ professional autonomy into the clinical care process; (2) nurse managers’ strategies for strengthening professional autonomy; and (3) balancing nurses’ professional autonomy with physician‐centered care in clinical practice. Nurse managers described professional autonomy as most visible in routine and individualized nursing care, particularly in complex clinical situations. Educational support, mentoring, participatory leadership, fair workload distribution, feedback, and a supportive organizational climate were identified as key facilitators. In contrast, physician‐centered organizational structures, hierarchical norms, heavy workloads, and limited resources were perceived as major constraints on nurses’ autonomous decision‐making.

**Conclusion:**

According to nurse managers, professional autonomy emerged as a dynamic process shaped by managerial practices, organizational conditions, and interprofessional relationships rather than solely an individual competence. Nurse managers play a critical role in fostering work environments that support nurses’ autonomous clinical judgment while maintaining safe and collaborative care.

**Implications for Nursing Management:**

Nurse managers should strengthen professional autonomy through structured orientation, competency‐based mentoring, participatory decision‐making, and equitable workload distribution. Creating psychologically safe environments that support open communication may enhance nurses’ autonomous judgment and improve outcomes.

## 1. Introduction

The roles and responsibilities of nurses have expanded, positioning them as key contributors within contemporary healthcare systems [1]. Healthcare organizations increasingly rely on nurses’ expertise and their capacity for independent clinical decision‐making to enhance the quality of care and service delivery [2]. Professional autonomy is a critical competency that enables nurses to make independent clinical decisions based on their clinical knowledge and experience and to manage care processes within the framework of professional responsibility [1]. Today, hospitals face multifaceted challenges, including emerging disease patterns, rapidly advancing medical technologies, a growing aging population, and ongoing budget constraints. These challenges necessitate that nurses continuously develop new knowledge and skills in order to adapt effectively to changes in healthcare delivery [3]. In this context, nurses’ professional autonomy has become an increasingly important strategic issue at the international level, particularly in relation to patient safety, quality of care, strengthening the nursing workforce, and the development of sustainable healthcare systems (International Council of Nurses [4]). Therefore, the ability of nurses to make independent clinical decisions and assume responsibility for those decisions is regarded as one of the fundamental components of contemporary nursing practice.

However, the literature indicates that nurses’ professional autonomy is not consistently realized at the expected level in clinical practice and that their organizational commitment, participatory roles, and professional contributions often remain insufficiently visible [5, 6]. Multiple individual and structural factors have been identified as constraining professional autonomy, including limitations in nursing education, role ambiguity, rigid organizational cultures, legal and ethical boundaries, physician‐centered organizational structures, lack of institutional support, and low participation in professional organizations [7–9]. These conditions may hinder nurses’ ability to exercise independent clinical judgment during the care process [10]. Professional autonomy in nursing can be conceptualized as a multidimensional process shaped by organizational structure, leadership approaches, and managerial support [1]. Accordingly, the creation of work environments in which nurses can freely apply clinical judgment, access learning and professional development opportunities, act autonomously, and receive managerial support is of critical importance [2, 11]. Empowering leadership approaches adopted by nurse managers that actively promote professional autonomy have been shown to facilitate nurses’ ability to meet job demands [11] and to support the adoption of advanced professional roles [12].

Previous research has primarily focused on staff nurses and has largely employed quantitative designs to examine the levels, determinants, and outcomes of professional autonomy. These studies have evaluated the effects of professional autonomy on quality of care, patient safety, job satisfaction, organizational commitment, and professional role expectations [1, 6, 13–15]. In addition to this body of research, Pursio et al. [2] explored nurse managers’ perceptions of nurses’ professional autonomy and the ways in which it is supported in clinical practice. Their findings highlighted the important role of nurse managers in strengthening professional autonomy, supporting professional development and expertise, facilitating access to advanced education, and fostering supportive work environments that encourage equal participation. Nevertheless, evidence remains limited regarding how nurse managers perceive the manifestation of professional autonomy in everyday clinical care and how they understand the managerial and organizational factors that facilitate or constrain autonomous nursing practice.

This study explores nurse managers’ perceptions of professional autonomy, how autonomy is reflected in clinical care practice, and the managerial and organizational dynamics that shape autonomous nursing practice. Understanding the perspectives of nurse managers is important, as they play a pivotal role in shaping organizational structures, work environments, and professional collaboration processes that support nurses’ professional autonomy [7]. Accordingly, the aim of this study was to examine nurse managers’ perceptions of nurses’ professional autonomy, how professional autonomy is supported within clinical care settings, and the managerial and organizational processes influencing autonomous nursing practice.

## 2. Background

Professional autonomy in nursing refers to nurses’ capacity to make clinical decisions, initiate appropriate interventions based on their professional knowledge and experience, and independently manage patient care within legal, ethical, and professional boundaries [7, 8, 10, 14, 16, 17]. It also encompasses contributing to the development of care standards, promoting patient safety, and improving work environments in which healthcare services are delivered [15, 16]. In this respect, professional autonomy is considered a fundamental component of nursing practice and an important determinant of both professional development and quality of care.

The development of professional autonomy is shaped by the interaction of individual competencies, organizational structures, interprofessional collaboration, and leadership practices. Research has demonstrated that higher levels of professional autonomy are associated with positive patient outcomes, including improved quality of care, a stronger patient safety culture, and lower rates of complications and mortality [13, 17, 18]. Professional autonomy has also been linked to a range of professional outcomes, such as enhanced job performance, organizational commitment, psychological empowerment, patient advocacy, professional competence, and nurse–physician collaboration [1, 8, 9, 14, 16, 19, 20]. Furthermore, supportive organizational cultures, shared leadership, participation in managerial processes, and regular feedback mechanisms have been identified as important factors that strengthen autonomous practice [6, 16, 21]. In this context, nurse managers play a crucial role in fostering professional autonomy by adopting supportive leadership approaches, promoting professional development, and creating work environments that facilitate autonomous nursing practice [2, 17, 22].

Although previous studies have provided important insights into professional autonomy in nursing, the existing literature has largely focused on bedside nurses’ individual experiences, work environments, leadership characteristics, and outcome variables such as patient safety [1, 6, 13–15]. In contrast, less attention has been given to how nurse managers perceive professional autonomy within clinical care processes and the managerial and organizational factors that influence its enactment in practice [2]. Examining professional autonomy from the perspective of nurse managers may enhance our understanding of how organizational structures, leadership practices, and interprofessional dynamics shape autonomous nursing practice. Therefore, exploring nurse managers’ views may provide valuable insights into the manifestation of professional autonomy in clinical settings and the organizational processes that support or constrain autonomous nursing practice. Accordingly, this study focuses on nurse managers’ perceptions of professional autonomy, its manifestation in clinical care, and the managerial and organizational factors influencing autonomous nursing practice.

To guide the study, the following research questions were developed: (1) How do nurse managers perceive nurses’ professional autonomy in clinical practice? (2) What strategies do nurse managers use to support and strengthen professional autonomy? (3) What managerial, organizational, and interprofessional factors influence the enactment of professional autonomy in clinical care settings?

## 3. Methods

### 3.1. Design

A qualitative descriptive design was used in this study. Qualitative description is an appropriate approach for obtaining a rich and straightforward account of participants’ perceptions, experiences, and perspectives within their natural context [23]. Rather than developing an interpretive theory or exploring the essence of lived experiences, this study sought to describe nurse managers’ views and experiences regarding professional autonomy in clinical practice. Therefore, a qualitative descriptive design was considered the most appropriate methodological approach. The study was reported in accordance with the Consolidated Criteria for Reporting Qualitative Research (COREQ) guidelines [24].

### 3.2. Setting

The study was conducted between March 1 and May 1, 2025 in the acute care units of a 300‐bed public hospital in Türkiye. Data were collected from officially assigned nurse managers responsible for supervising nursing care and clinical operations in inpatient units providing direct patient care services. Inpatient care services comprised medical, surgical, and intensive care units and consisted of a total of 22 clinical units.

### 3.3. Participants

A maximum variation purposive sampling strategy was used to recruit nurse managers with diverse characteristics, including gender, age, educational background, clinical unit, and nursing experience, in order to capture a broad range of perspectives relevant to the study phenomenon [25]. The sample comprised 15 nurse managers representing 15 different clinical departments, including obstetrics and gynecology, endocrinology, general surgery, internal medicine, physical therapy and rehabilitation, chest diseases, neurology, cardiology, nephrology, general intensive care, coronary intensive care, orthopedics, urology, palliative care, and gastroenterology (Table 1). Nurse managers who met the inclusion criteria and voluntarily provided informed consent were included in the study. The inclusion criteria were having at least 1 year of managerial experience in inpatient units and working as a nurse manager with active responsibility for supervising nursing care and clinical operations. Nurses working in nonmanagerial roles were excluded from the study.

**TABLE 1:** Sociodemographic characteristics of the nurse managers.

Characteristic		*n*
Age	35–39	6
	40–44	7
	45–49	2

Gender	Male	5
	Female	10

Nursing experience	10–14 years	7
	15–19 years	4
	20–24 years	4

TOTAL		15

The sample size was guided by the principle of information richness and the aim of obtaining a comprehensive understanding of nurse managers’ perspectives on professional autonomy. Interviews were conducted until sufficient depth and variation were achieved to address the study aim. After 15 interviews, the data provided adequate breadth and depth across different clinical units, and no substantially new insights relevant to the study objectives emerged. Therefore, the sample was considered sufficient to support a rich descriptive understanding of the phenomenon under investigation [26]. Participants were coded as NM1 to NM15 to ensure confidentiality (Table 1). Although none of the participants had attended a formal certificate‐based training program on leadership or professional autonomy, all were formally appointed to nurse manager positions within their clinical units.

### 3.4. Data Collection

Data were collected through individual semistructured interviews conducted to explore nurse managers’ perceptions of nurses’ professional autonomy in clinical care. An interview guide consisting of ten open‐ended questions was developed based on the relevant literature. In the development of the interview guide, a qualitative exploratory approach was adopted, and semistructured individual interviews were employed to facilitate an in‐depth exploration of participants’ experiences, perceptions, and managerial responsibilities related to professional autonomy [27, 28]. No formal theoretical or conceptual framework guided this study. Instead, the interview guide was informed by the literature on professional autonomy in nursing, nurse managers’ leadership roles, professional support mechanisms, and clinical decision‐making processes [2, 3, 9, 29]. The interview questions were designed to explore perceptions of professional autonomy, strategies used to support autonomy, factors influencing autonomous practice, and interprofessional dynamics within clinical settings, thereby ensuring alignment with the study aim (Table 2).

**TABLE 2:** Interview questions.

How do you define nurses’ professional autonomy?
How does professional autonomy contribute to the provision of high‐quality nursing care?
In what ways does professional autonomy shape nurses’ sense of professionalism?
What strategies do nurse managers use to support and develop nurses’ professional autonomy?
What types of educational and professional development opportunities are provided to enhance nurses’ professional autonomy?
In what types of work environments or professional communities can nurses’ professional autonomy be effectively enacted?
How do communication and collaboration among nurses influence their professional autonomy?
What factors facilitate or constrain the development of nurses’ professional autonomy?
How are professional roles related to nurses’ and physicians’ autonomy enacted in clinical practice?
How can nurse managers advance nurses’ professional autonomy within nurse–physician collaboration?

Before data collection, institutional permission was obtained from the hospital administration, after which nurse managers working in the participating clinical units where the study would be conducted were informed about the research. Participants were identified through purposive sampling, and nurse managers who met the eligibility criteria were invited to participate voluntarily through face‐to‐face contact. Interview schedules were then arranged with the nurse managers who agreed to participate, based on mutual availability. Interviews were conducted during working hours in a quiet and private room within the hospital’s administrative units to ensure confidentiality and minimize interruptions. Prior to data collection, participants were informed about the purpose of the study, confidentiality principles, and the voluntary nature of participation, and both verbal and written informed consent were obtained. With participants’ consent, all interviews were audio‐recorded using a digital voice recorder, and field notes were taken to document key observations. Each interview was conducted by the research team and lasted approximately 30–45 min. The total duration of audio recordings obtained from all interviews was approximately 9 h, and the interviews were transcribed verbatim by the researchers, resulting in approximately 90 pages of textual data.

The characteristics of the researchers are presented below in accordance with the COREQ recommendations. The researchers had relevant field knowledge and clinical experience related to acute care nursing services and were trained and experienced in qualitative research methods. Their academic and clinical backgrounds facilitated an in‐depth exploration of the research questions from a nursing perspective while maintaining methodological rigor.

### 3.5. Data Analysis

**FIGURE 1:**
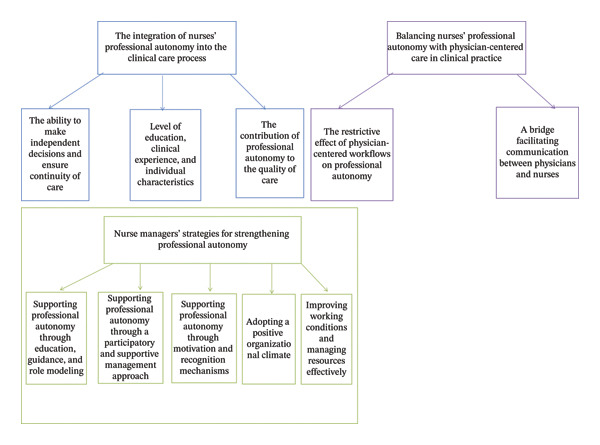
Nurse managers’ perspectives on the enactment and support of professional autonomy in nursing practice (source: authors’ own elaboration using MAXQDA 2024).

Data analysis was conducted using inductive qualitative content analysis in accordance with the COREQ guidelines. MAXQDA Analytics Pro 2024 software was used to systematically organize the data, support coding, and visualize relationships among codes and categories (Figure 1). The data were analyzed in accordance with the content analysis approach proposed by Graneheim and Lundman [30].

Following the framework proposed by Graneheim and Lundman [30], the analysis was conducted in five steps: (1) audio‐recorded interviews were transcribed verbatim; the transcripts were read repeatedly to gain an overall understanding, and analytic notes were taken during this process; (2) meaning units related to nurse managers’ views on nurses’ professional autonomy in clinical care were identified; (3) meaning units were reviewed to ensure that they contained sufficient information; (4) condensed meaning units were summarized and labeled with codes to represent their core content; and (5) codes were compared based on similarities and differences and grouped into categories. An example of the coding and category development process is presented in Table 3.

**TABLE 3:** An example of category structure.

Category	Subcategory	Codes	Illustrative quotations
The integration of nurses’ professional autonomy into the clinical care process	The ability to make independent decisions and ensure continuity of care Level of education, clinical experience, and individual characteristics The contribution of professional autonomy to the quality of care	Clinical decision‐making based on observation and assessment The nurse’s autonomous and holistic role Knowledge‐ and experience‐based clinical decision‐making Assuming responsibility for the outcomes Ethical decision‐making in the best interest of the patient The contribution of educational level to professional autonomy The impact of clinical experience on autonomy Individual differences in approach Increased patient satisfaction Improved patient recovery and reduced readmissions Ensuring patient safety through early intervention Increased nurse job satisfaction and motivation	“The patient had a pressure injury. We conduct daily assessments using pressure injury risk scales, reposition the patient regularly, and perform wound dressings.” (NM 3) “As nurses, we are at the patient’s bedside 24/7; we continuously monitor pain, bleeding, and vital signs and oversee every stage of the postoperative process. While the physician performs the surgery and leaves, we ensure continuity of the patient’s care.” (NM 1) “For example, when a patient’s condition deteriorates, initiating a code blue and intervening until the physician arrives can be life‐saving.” (NM 7) “Taking independent decisions in the care process and assuming responsibility for the implementation of those decisions.” (NM 6) “In the intensive care unit, the patient needs to be shaved. Even if the family objects, we proceed with shaving because it is an intervention carried out in the patient’s best interest.” (NM 10) “There is a difference between vocational high school–educated nurses and bachelor’s degree–educated nurses in terms of performing clinical practices consciously.” (NM 9) “The length of time spent working on the ward and the effort invested influence the development of professional autonomy.” (NM 7) “When a patient begins to experience pain, I try to distract the patient, whereas another nurse may suggest informing the physician.” (NM 12) “When nurses are autonomous and supported in making independent decisions, the quality of care improves, and both healthcare staff and patients experience greater satisfaction.” (NM 1) “When nurses provide education on diabetic wound care, patients’ wounds heal more quickly. I believe that providing discharge education also reduces readmissions.” (NM 3) “By performing appropriate interventions before reaching the physician, nurses can prevent harm to the patient.” (NM 5) When nurses are autonomous and supported in making independent decisions, the quality of care improves, and healthcare staff are happier and more motivated.” (NM1)

### 3.6. Rigor and Trustworthiness

Trustworthiness was ensured by addressing credibility, transferability, dependability, and confirmability throughout all stages of the research process [30]. The researchers’ clinical and academic experience in nursing care and management facilitated an in‐depth understanding of the research context. Reflexive practices were employed during data collection and analysis to minimize potential bias. The researchers were familiar with the clinical setting through their academic and clinical activities and had prior professional relationships with some of the nurse managers; however, several participants were not previously known to the researchers and were first approached during the recruitment process. The researchers had no managerial, supervisory, or evaluative authority over any of the participants. To minimize the potential influence of professional roles and power dynamics, participants were informed that participation was entirely voluntary, that all responses would remain confidential, and that declining participation or withdrawing from the study would have no consequences. Throughout data collection and analysis, the researchers engaged in reflexive discussions to critically examine how their professional backgrounds, prior assumptions, and familiarity with the clinical context might influence data interpretation. To strengthen credibility, coding and subcategory development were conducted independently by the researchers. Codes and subcategories were reviewed multiple times to ensure adequate coverage of the data, and similarities and differences among subcategories were carefully examined. The researchers subsequently compared, discussed, and reached consensus on the findings. To enhance transparency and allow readers to follow the analytic process, the relationships among codes, subcategories, and categories are presented in Table 3. In addition, representative verbatim quotations were included under each category to support the findings [31]. Transferability was supported by providing detailed descriptions of participants’ characteristics, settings, and experiences, enabling readers to assess the applicability of the findings to similar contexts. Interviews were transcribed verbatim, and direct quotations were used to illustrate participants’ perspectives. Dependability was ensured through the internal consistency of participants’ accounts within the defined study period and analytic framework. Finally, confirmability was addressed by clearly documenting how interpretations and conclusions were derived from the data [32]. Throughout the study, explicit attention was given to articulating the rationale for theoretical, methodological, and analytical decisions.

## 4. Results

The findings were organized into three main categories reflecting nurse managers’ perceptions of nurses’ professional autonomy, how professional autonomy is supported within clinical care settings, and the managerial and organizational processes influencing autonomous nursing practice.

a. The integration of nurses’ professional autonomy into the clinical care process
b. Nurse managers’ strategies for strengthening professional autonomy
c. Balancing nurses’ professional autonomy with physician‐centered care in clinical practice.

**FIGURE 2:**
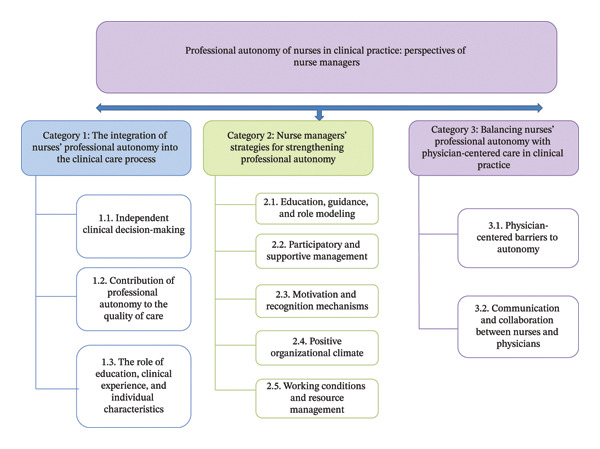
Overview of categories and subcategories describing nurse managers’ perceptions of professional autonomy in nursing practice.

The categories and subcategories identified through the analysis are presented in Figure 2. A detailed thematic structure, including categories, subcategories, codes, and representative quotations, is provided in Supporting File 1.

### 4.1. The Integration of Nurses’ Professional Autonomy Into the Clinical Care Process

In this study, nurse managers described nurses as being able to make independent, ethically grounded decisions based on clinical observation, assessment, and clearly defined professional roles, with a primary focus on patients’ conditions and best interests. Nurse managers emphasized that nurses integrate these decisions into the care process and assume responsibility for the outcomes of their interventions. Nurse managers described nurses as having a central role—who remain in continuous contact with patients and monitor every stage of the postoperative process—in planning and sustaining patient care. Nurse managers described nurses’ ability to intervene appropriately in emergency situations until a physician arrives, to make critical decisions by assessing ventilator functions, and to independently manage observation, assessment, and care planning related to aspiration risk, pressure injuries, and infection prevention as concrete manifestations of professional autonomy in clinical practice. According to nurse managers, nonpharmacological pain management and patient education were identified as key professional responsibilities through which nurses exercise autonomous clinical judgment. The following quotations from nurse managers illustrate these experiences:

“As nurses, we are at the patient’s bedside 24/7… Physicians perform the surgery and leave, but we ensure the continuity of patient care. This demonstrates the nurse’s autonomous and holistic role within the clinical process” [NM 1].

*“*For example, when a patient’s condition deteriorates, activating a Code Blue and intervening until the physician arrives can save the patient’s life. Nurses can perform aspiration and maintain airway patency, thereby sustaining the patient’s vital functions.” [NM 7].

“The patient had a pressure injury. We assess the patient daily using pressure injury risk scales, use air mattresses, reposition the patient, and perform wound dressings. We do not ask the physician about everything. Physicians are often in the operating room; we know what they expect, and they know us. They even thank us for our work” [NM 3].

According to nurse managers, nurses’ educational background, clinical experience, and individual characteristics were important factors influencing the development of professional autonomy. Nurse managers reported that nurses with a bachelor’s degree and experience across different clinical units were better able to assess clinical situations holistically and demonstrated stronger independent decision‐making skills. In contrast, nurse managers perceived that nurses who had worked for prolonged periods within the same unit experienced greater difficulty in approaching diverse clinical situations from an autonomous perspective. In addition, professional awareness and a strong sense of individual responsibility were described by nurse managers as factors that enhance nurses’ capacity to take initiative and make independent care‐related decisions in clinical practice. The following quotations illustrate nurse managers’ views on the role of education, experience, and individual differences in shaping professional autonomy:

“Experienced nurses already know to apply cold therapy to a patient with phlebitis. However, for newly employed nurses, professional autonomy is critically important for developing clinical decision‐making skills and assuming responsibility for independent practice.” [NM 4].

“Nurses can approach the same situation in different ways. When a patient starts to experience pain, I may try to distract the patient, whereas another nurse may suggest informing the physician. This shows how nurses exercise their professional autonomy, and it can vary from one nurse to another.” [NM 12].

According to participants, professional autonomy was perceived to contribute substantially to effective care delivery by enabling nurses to take initiative in clinical assessment, care planning, and intervention processes. Nurse managers emphasized that autonomous nurses were perceived to facilitate faster patient recovery, enhance the quality of care, and support the delivery of holistic and continuous nursing care. A supportive work environment was perceived to strengthen nurses’ professional autonomy, which nurse managers believed increased motivation and professional satisfaction and positively influenced patient care. The following quotations illustrate nurse managers’ perspectives on the contribution of professional autonomy to care quality:

“The healing of pressure injuries, nutrition, bathing, and overall care depend on how regularly and carefully these are carried out by nurses. During the intubation process, if the nurse performs aspiration monitoring effectively, the patient can be extubated more quickly. In fact, patient recovery largely depends on how effectively the nurse applies knowledge, skills, and a sense of ethical responsibility” [NM 11].

### 4.2. Nurse Managers’ Strategies for Strengthening Professional Autonomy

The findings suggest that nurse managers reported employing educational, guidance‐based, and role‐modeling strategies to support and enhance nurses’ professional autonomy. Nurse managers reported strengthening nurses’ knowledge, skills, and decision‐making capacities through in‐service training, certification programs, and structured orientation processes. In addition, autonomy was perceived to be supported through coaching and one‐to‐one guidance during clinical practice. The involvement of experienced nurses in mentoring newly employed nurses was described as facilitating learning processes and supporting the development of clinical decision‐making skills. The following quotations illustrate nurse managers’ experiences in supporting and developing professional autonomy:

“I lead in a way that supports professional autonomy by considering my staff’s levels of knowledge, skills, and expertise. Some nurses are more inclined toward certain tasks, while others may need additional support. Nurses with limited experience already know what to do, but for those with less experience, I work one‐to‐one, demonstrate clinical practices, provide guidance, and try to prepare them to make independent decisions.” [NM 5].

Nurse managers reported adopting a management approach that encourages nurses’ participation in decision‐making processes, provides a work environment in which nurses can freely express their views, strengthens effective communication and collaboration within teams, and emphasizes learning from errors. This participatory and supportive management style was described as a key strategy for enhancing professional autonomy by empowering nurses to voice their opinions, take initiative, and actively engage in teamwork. Participants perceived that such an environment was perceived to foster motivation and job satisfaction among nurses, which in turn positively influences the quality of patient care. The following quotations illustrate nurse managers’ experiences with participatory and supportive management practices:

“We had a nurse whose nebulizer frequently malfunctioned. She suggested using a double‐sided jug as a solution and felt comfortable sharing this idea with me. As the charge nurse, I presented it to the management. A trial was conducted, and the need to carry nebulizers was eliminated. Now, double‐sided jugs are used throughout the unit. This was possible because nurses felt free to communicate openly. In such a work environment, nurses’ motivation increases, job satisfaction improves, and this is positively reflected in patient care.” [NM 11].

Nurse managers reported supporting professional autonomy through motivation and recognition mechanisms, including fair workload planning, developmental feedback, reward and recognition practices, and activities designed to enhance team motivation. Participants emphasized that making achievements visible within the team strengthens solidarity and a sense of belonging, while equitable task distribution and acknowledgment of effort enhance nurses’ job satisfaction. These practices were described as reinforcing nurses’ willingness to take initiative and engage autonomously in clinical decision‐making. The following quotations illustrate nurse managers’ experiences with motivation‐ and recognition‐based strategies:

“I arrange duty schedules, weekly and monthly rosters, and payroll records in a fair and equitable manner. Each physician has assigned nurses, and sometimes one nurse may be responsible for two patients while another has seven or eight. On days when a physician has surgery, that nurse may be very busy. In such cases, other nurses support their colleague—while one nurse administers treatment, another provides preoperative care for the busy nurse’s patients. In this way, communication and collaboration within the team are strengthened.” [NM 13].

Nurse managers identified the creation of a positive and psychologically safe organizational climate as a key strategy for supporting and enhancing professional autonomy. Professional autonomy was perceived to develop more effectively in work environments where nurses feel safe, valued, and institutionally supported. Participants emphasized that open communication, mutual trust, teamwork, and a workplace culture free from mobbing strengthen nurses’ ability to make independent decisions and enhance their professional confidence. In addition, nurse managers highlighted that a fair and democratic organizational culture—characterized by clearly defined authority and responsibility boundaries and supported by both nurse managers and physicians—contributes to increased professional autonomy. Such environments were described as fostering professional development, job satisfaction, and organizational commitment, while reinforcing nurses’ perception of being equal members of the healthcare team rather than subordinate assistants. The following quotations illustrate nurse managers’ views on the importance of a positive and safe organizational climate:

“There should be an environment in which certain authorities are granted to nurses. If a patient is in pain, I should be able to use my professional autonomy. Some rules should also apply to nurses, and their scope of authority should be expanded. There should be a free working environment where nurses are not seen as physicians’ assistants but as full members of the team.” [NM 5].

Nurse managers reported focusing on improving working conditions and managing resources effectively as a key strategy for supporting and developing professional autonomy. Ensuring the availability of adequate and high‐quality equipment, planning sufficient nurse staffing levels, and maintaining a balanced workload were emphasized as factors perceived to strengthen nurses’ ability to exercise professional autonomy and independent clinical decision‐making. In contrast, high patient acuity, staffing shortages, and limited resources were perceived as constraining professional autonomy and negatively affecting the quality of care. The following quotations illustrate nurse managers’ experiences regarding working conditions and resource management:

“The patient load and the number of nurses must be proportional. The workload should not be excessive—sometimes even on weekends, two nurses are responsible for 20 patients. Can autonomy exist in such a unit? We are just trying to keep up with the work. The workload is extremely heavy. All patients are in the same unit; some could be transferred out to reduce our workload.” [NM 7].

“In the intensive care unit, I request devices and materials. When a patient has a wound and there are insufficient supplies, what can a nurse do? They may only apply antiseptic and cover it. To provide effective care, I ensure the availability of materials, oral care solutions, disinfectants, and ICU‐specific supplies. Having complete and adequate equipment is essential for nurses to perform clinical interventions. When sufficient and high‐quality materials are available, nurses can implement their own clinical decisions.” [NM 10].

### 4.3. Balancing Nurses’ Professional Autonomy With Physician‐Centered Care in Clinical Practice

Despite nurses’ autonomous and holistic roles in routine care and individualized clinical practices, some nurse managers reported that physician‐centered workflows limit nurses’ professional autonomy in clinical decision‐making processes. Physicians’ preferences and working styles were described as frequently constraining nurses’ own clinical decisions and practices. On the other hand, in emergency and critical situations, nurses’ need to make rapid decisions creates tension between their responsibility for patient safety and physician‐centered workflows.

“In clinical practice, what the physician says is generally followed. Even when I make a suggestion for a patient with fever, I may receive harsh reactions such as, ‘Are you the one making the decision?’ This situation restricts nurses’ autonomy in clinical decision‐making processes. However, physicians could communicate and collaborate with nurses.” [NM 5].

“In some situations, we are required to make rapid decisions about the patient when a physician is not immediately available. For example, a patient may remove their catheter and cause self‐harm. In such cases, it may be necessary to apply restraints to ensure the safety of an agitated patient; however, obtaining physician approval is not always feasible. Subsequently, complications such as circulatory impairment may occur, and the physician may respond by stating, ‘Do not apply restraints without consulting me.’ These situations place us in a dilemma between our responsibility to protect the patient and the constraints of a physician‐centered system.” [NM 14].

Participants reported that nurses’ autonomous and holistic care roles were perceived to develop more readily in environments where effective communication and collaboration with physicians are established. Nurse managers were described as acting as a bridge between physicians and nurses by facilitating communication processes, helping nurses clarify their professional roles, and supporting the delivery of care within the framework of nursing responsibility in collaboration with physicians. According to participants, strengthening professional autonomy was perceived to require clearly defined nurse and physician roles, respect for each profession’s scope of responsibility, and making nurses’ knowledge and practical competencies more visible.

“In care practices, for example in interventions such as cold application or massage, physicians generally do not interfere. So, in some areas we are independent, but the overall functioning is still physician‐centered.” [NM 7].

“In clinical practice, there is generally a physician‐centered workflow; however, as the charge nurse, I pay attention to maintaining this balance. I try to strengthen communication between physicians and nurses and ensure that care practices remain within nurses’ areas of responsibility.” [NM 9].

## 5. Discussion

### 5.1. The Integration of Nurses’ Professional Autonomy Into the Clinical Care Process

Professional autonomy in nursing is defined as an important professional competence that enables nurses to assess patients’ needs, make independent decisions, and initiate appropriate care interventions in accordance with their professional competencies [8, 16, 33]. In the present study, nurse managers reported that professional autonomy becomes particularly visible in clinical practice through nurses’ assessment of patients, their ability to make independent and ethically grounded decisions, integrate these decisions into the care process, and assume responsibility for their outcomes. This finding may be related to nurses’ continuous contact with patients and their ongoing monitoring of the care process. In particular, the responsibility for rapid assessment and decision‐making during postoperative care, emergency situations, and routine patient monitoring may increase the visibility of professional autonomy in clinical practice. Consistent with this finding, previous studies have shown that professional autonomy is more evident in settings characterized by a high burden of clinical decision‐making [2, 8, 14]. Furthermore, professional responsibility in nursing encompasses not only the delivery of care but also the justification of clinical decisions and accountability for their outcomes [12].

In the present study, nurse managers identified educational level, clinical experience, and a sense of individual responsibility as important factors influencing the development of professional autonomy. In particular, experience gained across different clinical settings may contribute to nurses’ ability to evaluate clinical situations more holistically and to develop stronger independent decision‐making skills. In addition, higher educational attainment and increased professional awareness may support nurses in taking greater initiative in clinical practice. These findings are consistent with previous studies reporting that education, professional experience, and self‐confidence strengthen nurses’ clinical competence and autonomous decision‐making abilities [1, 2, 7, 17, 33, 34].

Professional autonomy has been reported to have positive effects on patient safety, quality of care, and nurses’ participation in decision‐making processes [2, 6, 35]. Similarly, nurse managers in the present study reported that autonomous nursing practices support timely interventions and effective care delivery. This may be related to nurses’ position as the healthcare professionals closest to patients, enabling them to recognize changing care needs early and initiate appropriate interventions. In addition, supportive work environments may enable nurses to exercise their professional autonomy more effectively, thereby contributing positively to motivation, job satisfaction, and quality of care. Consistent with these findings, previous studies have shown that supporting professional autonomy increases nurses’ active participation in decision‐making processes, strengthens collaboration within multidisciplinary teams, and enhances work motivation, professional commitment, and professional pride, ultimately contributing to improved quality of patient care [1, 2, 6, 17].

### 5.2. Nurse Managers’ Strategies for Strengthening Professional Autonomy

In the present study, nurse managers emphasized the importance of educational leadership strategies, including education and certification programs, structured orientation processes, guidance and mentoring practices, and role modeling, in supporting the development of professional autonomy. This may be explained by the greater need for support among nurses with limited clinical experience during independent decision‐making processes. Participants also indicated that guidance tailored to nurses’ levels of knowledge, skills, and expertise contributes to the development of their independent decision‐making capacity. These findings are consistent with previous studies reporting that professional competence, knowledge, skills, expertise, and leadership characteristics are key determinants of nurses’ professional autonomy [2, 29]. Therefore, it is important for nurse managers to provide educational opportunities that support professional development, foster a collaborative work culture, and create learning opportunities that strengthen leadership and self‐management skills [36, 37].

Another important finding was that nurse managers adopted participatory and empowering management approaches to support professional autonomy. Encouraging participation in decision‐making processes, creating open and trust‐based communication environments, and strengthening teamwork were identified as key factors that enhance nurses’ perceptions of autonomy. This may reflect nurses’ greater willingness to take initiative in work environments where they can freely express their views and feel supported within managerial processes. These findings are consistent with studies showing that nurses prefer decision‐making processes in which their opinions are valued and their active participation is encouraged [33]. Similarly, managerial and structural opportunities that promote nurses’ active involvement in decision‐making have been identified as important for the sustainability of professional autonomy [38, 39]. In addition, strengthening interdisciplinary collaboration, ensuring that managers are visible and accessible in clinical settings, demonstrating effective leadership, and encouraging information sharing have been reported as practices that support the development of professional autonomy [37, 39, 40].

In the present study, nurse managers emphasized the importance of the work environment and organizational resources in supporting the development of professional autonomy. Adequate staffing, appropriate equipment, favorable working conditions, access to high‐quality materials, and balanced nurse–patient ratios were reported to facilitate nurses’ ability to implement independent clinical decisions. In contrast, heavy workloads, staffing shortages, and organizational constraints were identified as factors that limit professional autonomy. These findings are consistent with previous studies reporting that intensive and stressful work environments, managerial constraints, and role ambiguity weaken professional autonomy [13, 16, 29, 41]. Likewise, nurses working in supportive and well‐structured environments have been reported to focus more effectively on patient care and implement autonomous decisions more efficiently [14]. Therefore, it is critical for nurse managers to support favorable working conditions, promote training initiatives that strengthen teamwork, and foster a collaborative organizational culture [38, 39].

### 5.3. Balancing Nurses’ Professional Autonomy With Physician‐Centered Care in Clinical Practice

In the present study, nurse managers reported that nurses’ areas of professional autonomy are constrained in clinical environments where physician‐centered decision‐making processes predominate. In particular, they noted that tensions may arise between nurses’ responsibility to make rapid decisions in emergency and critical situations and physician‐centered expectations. Although diagnosis, treatment planning, and medication management fall within physicians’ responsibilities, professional nursing autonomy is expected to encompass practices grounded in nursing knowledge, skills, and clinical judgment [2, 16]. Some nurse managers indicated that physicians occasionally adopt directive and restrictive attitudes even in areas of care that should be performed independently by nurses. Possible explanations for this situation include hierarchical organizational structures, authoritarian management approaches, and unclear organizational rules [16]. Similarly, previous studies have shown that physicians may restrict nurses’ use of initiative even in areas of independent nursing practice, such as nonpharmacological pain management [29]. Such structural barriers may negatively affect nurses’ ability to effectively utilize their professional knowledge and experience, reduce their motivation, limit their visibility within multidisciplinary teams, and hinder their contributions to patient care processes [16, 33].

Another important finding was that nurse managers assumed a bridging role in strengthening collaboration between nurses and physicians. Participants indicated that clarifying professional boundaries, facilitating effective communication, and promoting team‐based collaboration contribute to the strengthening of professional autonomy. This finding is consistent with previous studies demonstrating that positive nurse–physician collaboration supports professional autonomy [2, 19]. Furthermore, shared patient rounds and joint decision‐making processes have been reported to enhance both interprofessional collaboration and professional autonomy [29]. Similarly, in the present study, nurse managers’ efforts to increase the visibility of nursing care appeared to contribute to physicians’ better understanding of nurses’ roles in clinical decision‐making processes. This finding is also in line with studies showing that leadership approaches that support interprofessional teamwork play a critical role in fostering positive organizational cultures and are associated with improved patient outcomes and greater job satisfaction [16, 42]. In this context, strengthening interdisciplinary respect, professional self‐awareness, and collaborative skills, while emphasizing the complementary contributions of different disciplines and supporting the development of a shared organizational vision, may further promote both professional autonomy and interprofessional collaboration [42].

Overall, the findings suggest that nurse managers perceive nurses as playing a holistic role in clinical care processes and as being better able to implement independent care decisions when appropriate organizational conditions are in place. In this regard, a trust‐based and supportive organizational culture that promotes autonomy appears to be important for strengthening nurses’ professional roles and enhancing the effectiveness of care delivery. The findings further indicate that, in addition to managerial leadership, organizational structures and interdisciplinary collaboration play a critical role in the sustainable support of professional autonomy. Nurse managers were also perceived to serve as important mediators in balancing nurses’ autonomous care practices with physician‐centered clinical systems. In this respect, the study contributes a managerial and organizational perspective to the ongoing discussion on professional autonomy.

### 5.4. Limitations of the Study

This study has several limitations. First, because the study was conducted in a public hospital and advanced professional nursing practice models were not systematically implemented within the institution, it may limit the generalizability of the findings. This situation requires cautious interpretation of the results, particularly regarding conclusions about how organizational structures shape professional autonomy. Second, the fact that nurse managers had not received systematic training in leadership or professional autonomy may have limited their evaluations and interpretations of the autonomy concept. Third, as the study was carried out in a single institution, organizational structure–specific characteristics may have influenced the findings; therefore, the results should not be directly generalized to different types of healthcare institutions. Finally, since the study relied solely on the perspectives of nurse managers, the professional autonomy experiences of bedside nurses could not be directly captured. In addition, the collection of data through self‐reported individual interviews may have led participants to provide socially desirable responses due to their managerial roles. Furthermore, although the researchers’ academic and clinical experience in nursing contributed to the data interpretation process, researcher interpretation bias, which is inherent in qualitative research, may have influenced the findings. Therefore, the contextual and interpretive nature of the study should be taken into consideration when evaluating the findings.

## 6. Conclusion

The findings of this study provide insight into nurse managers’ perceptions of bedside nurses’ professional autonomy and suggest that autonomy may be influenced by both individual competencies and organizational and managerial factors. The findings were organized into three interrelated categories: (a) the integration of nurses’ professional autonomy into the clinical care process; (b) nurse managers’ strategies for supporting and developing professional autonomy; and (c) balancing nurses’ professional autonomy with physician‐centered care in clinical practice.

Within the context of this study, nurse managers described several challenges that may influence the development of professional autonomy, including physician‐centered structures, heavy workloads, limited resources, and organizational constraints. At the same time, participants emphasized the potential importance of managerial support, professional development opportunities, participatory management approaches, recognition mechanisms, and effective physician–nurse communication in facilitating autonomous nursing practice.

The findings suggest that supporting professional autonomy may require not only individual efforts by nurses but also organizational and managerial initiatives. Accordingly, healthcare organizations may benefit from creating environments that encourage nurses’ participation in decision‐making processes, support professional development, and promote collaborative interprofessional relationships. Given the context‐specific nature of this study and its relatively small sample, further research involving different institutions, healthcare settings, and participant groups is needed to explore these issues in greater depth. Future studies may also examine how organizational characteristics, including magnet hospital principles, positive work environments, and leadership interventions, influence the development and sustainability of professional autonomy in nursing practice.

## 7. Implications for Nursing Management

Nurse managers should systematically implement structured orientation programs, continuous professional development opportunities, and mentoring systems tailored to nurses’ educational levels and clinical experience. Clarifying professional roles, defining interprofessional boundaries, and fostering mutual respect within the healthcare team may contribute to reducing hierarchical barriers that limit nurses’ autonomous decision‐making processes. In addition, managerial advocacy for adequate staffing levels, balanced workload distribution, and the provision of necessary material resources is critical to enabling nurses to effectively exercise their autonomous clinical judgment. Furthermore, establishing an organizational climate that is trust‐based, supports open communication, and adopts a nonpunitive approach to errors will strengthen autonomous and responsibility‐based nursing practices. Nurse managers who adopt a holistic approach integrating autonomy‐supportive leadership, effective resource management, and interprofessional collaboration may help improve the quality of nursing practice, patient outcomes, and overall organizational performance.

## Author Contributions

G.Ö.Ö. conceived and designed the study, collected and analyzed the data, interpreted the findings, and drafted the manuscript. R.Y. contributed to data collection, data analysis, interpretation of the findings, and critical revision of the manuscript.

## Funding

This research received no specific grant from any funding agency in the public, commercial, or not‐for‐profit sectors.

## Disclosure

All the authors read and approved the final manuscript.

## Ethics Statement

Ethical approval for this study was obtained from the Ethics Committee of Cankiri Karatekin University (Approval No: e4fceb0ff55045c9; January 29, 2025). Institutional permissions were subsequently obtained from the hospital chief physician and the Provincial Health Directorate (Permission No: E‐64943697‐799‐268853177, February 18, 2025). Before data collection, all nurse managers were informed about the purpose of the study, the voluntary nature of participation, confidentiality, and their right to withdraw at any time without any consequences. Written and verbal informed consent was obtained from all participants. Participants were also informed that the interviews would be audio‐recorded, and separate consent for audio recording was obtained prior to the interviews.

## Conflicts of Interest

The authors declare no conflicts of interest.

## Supporting Information

Additional supporting information can be found online in the Supporting Information section.

## Supporting information


**Supporting Information** Supporting File 1: Thematic structure including categories, subcategories, codes, and sample quotations.

## Data Availability

The datasets generated and/or analyzed during the current study are not publicly available due to ethical and confidentiality restrictions but are available from the corresponding author on reasonable request.
