# Supplementary material for: Professional Autonomy in Nursing Practice: A Qualitative Study From the Perspective of Nurse Managers
Source: J Nurs Manag. 2026 Jul 21;2026:4380993. doi: 10.1155/jonm/4380993 (PMC13386666; doi:10.1155/jonm/4380993)
Supplement: Supplementary file 1 — Supporting Information Supporting File 1: Thematic structure including categories, subcategories, codes, and sample quotations. [file JONM-2026-4380993-s001.docx]

**Supplementary File 1:** Example of category development including categories, subcategories, codes, and illustrative quotations

| **Category** | **Sub-category** | **Codes** | **Illustrative** **quotations** |
| --- | --- | --- | --- |
| The integration of nurses’ professional autonomy into the clinical care process | The ability to make independent decisions and ensure continuity of care  Level of education, clinical experience, and individual characteristics  The contribution of professional autonomy to the quality of care | Clinical decision-making based on observation and assessment  The nurse’s autonomous and holistic role  Knowledge- and experience-based clinical decision-making  Assuming responsibility for the outcomes  Ethical decision-making in the best interest of the patient  The contribution of educational level to professional autonomy  The impact of clinical experience on autonomy  Individual differences in approach  Increased patient satisfaction  Improved patient recovery and reduced readmissions  Ensuring patient safety through early intervention  Increased nurse job satisfaction and motivation | “The patient had a pressure injury. We conduct daily assessments using pressure injury risk scales, reposition the patient regularly, and perform wound dressings.” (NM 3)  “As nurses, we are at the patient’s bedside 24/7; we continuously monitor pain, bleeding, and vital signs and oversee every stage of the postoperative process. While the physician performs the surgery and leaves, we ensure continuity of the patient’s care.” (NM 1)  “For example, when a patient’s condition deteriorates, initiating a code blue and intervening until the physician arrives can be life-saving.” (NM 7)  “Taking independent decisions in the care process and assuming responsibility for the implementation of those decisions.” (NM 6)  “In the intensive care unit, the patient needs to be shaved. Even if the family objects, we proceed with shaving because it is an intervention carried out in the patient’s best interest.” (NM 10)  “There is a difference between vocational high school–educated nurses and bachelor’s degree–educated nurses in terms of performing clinical practices consciously.” (NM 9)  “The length of time spent working on the ward and the effort invested influence the development of professional autonomy.” (NM 7)  “When a patient begins to experience pain, I try to distract the patient, whereas another nurse may suggest informing the physician.” (NM 12)  “When nurses are autonomous and supported in making independent decisions, the quality of care improves, and both healthcare staff and patients experience greater satisfaction.” (NM 1)  “When nurses provide education on diabetic wound care, patients’ wounds heal more quickly. I believe that providing discharge education also reduces readmissions.” (NM 3)  “By performing appropriate interventions before reaching the physician, nurses can prevent harm to the patient.” (NM 5)  When nurses are autonomous and supported in making independent decisions, the quality of care improves, and healthcare staff are happier and more motivated.” (NM1) |
| Nurse managers’ strategies for strengthening professional autonomy | Supporting professional autonomy through education, guidance, and role modeling  Supporting professional autonomy through a participatory and supportive management approach  Supporting professional autonomy through motivation and recognition mechanisms  Adopting a positive organizational climate  Improving working conditions and managing resources effectively | Role modeling and a coaching approach  Supporting in-service training and certification programs  Orientation and one-to-one mentoring practices  Considering nurses’ levels of knowledge and skills  Encouraging participation in decision-making processes  Creating an open communication environment  Strengthening team cohesion  Empowering leadership and morale enhancement  Supporting nurses in conscience-based decisions  Prioritizing collaboration over an authority-based approach  Fair workload planning  Reward and recognition  Organizing activities to enhance team motivation  Supporting development through feedback  A safe and supportive work environment  A safe work environment free from mobbing  An expanding scope of autonomous practice  Collaboration and teamwork  Ensuring the availability of adequate and high-quality materials  The use of innovative and modern equipment  Institutional comfort and the working environment  Workload and nurse-to-patient ratio imbalance | “I try to support nurses’ autonomy by serving as a role model and providing coaching in the clinical setting. I also enter the clinical area with nurses and perform patient care practices to be an exemplary nurse.” (NM 1)  **“I send nurses to intensive care certification programs. Every month, the nurse educator shares the training schedule with us, and I direct my staff accordingly.” (NM 2)** **“We participate in in-service training, and I also organize working conditions to encourage nurses to pursue further education.” (NM10)**  **“I support newly hired nurses during orientation training by providing guidance on patient care and professional autonomy.” (NM 4)** **“We assign newly employed nurses to work alongside experienced nurses until they fully adapt to the clinical environment.” (NM 8)**  “I lead in a way that supports nurses’ professional autonomy by considering their levels of knowledge and skills. I work one-to-one with less experienced nurses and demonstrate clinical practices.” (NM 5)  “I listen to my nurses and involve them in the decision-making process by incorporating their knowledge and experience.” (NM 6)  “Nurse managers should create a work environment in which they communicate and collaborate with one another, allowing nurses the freedom to express themselves.” (NM 6)  “When two nurses work well together, they first bathe one patient and then jointly care for the other. I motivate them by acknowledging that they have performed this practice very well.” (NM8, NM12)  “Even when mistakes occur, I defend them. I support my staff and stand by them.” (NM 7)  “When there is no written order while a patient is in pain, our nurses may intervene to prevent the patient from suffering. I support their conscience-based decisions.” (NM 6)  “I do not constantly exert pressure or establish authority; instead, I foster an environment of collaboration.” (NM12)  “I arrange duty schedules, weekly and monthly work rosters, and payroll records in a fair and equitable manner.” (NM 13)  “Each month, I submit the names of three nurses to the chief physician. We provide financial incentives from the revolving fund to increase their motivation.” (NM 4)  “I celebrate birthdays by buying cake and organizing small gestures such as chocolate and coffee, ensuring that these activities are planned inclusively without favoritism.” (NM 10)  “I participate in clinical rounds, observe my nurses, and provide constructive feedback when necessary.” (NM 3)  “It takes place in a work environment where nurses feel safe and valued, and where open communication, collaboration, and mutual trust are present.” (NM 1)  “Nurses should know which physicians will not react negatively; some physicians may question interventions such as asking why cold therapy was applied to their patient. We need understanding physicians.” (NM 4)  “An environment in which certain authorities are also granted to nurses, their responsibilities are expanded, and they are able to work freely with broader professional autonomy.” (NM 5)  “Effective teamwork is essential. For this, nurses and physicians need to communicate well and jointly assess the patient.” (NM 8–9)  **“During the pandemic, we never experienced shortages of supplies; every time we entered a room, we frequently changed gloves and disposable gowns.” (NM 6). “In the intensive care unit, I request equipment and materials. Having complete and adequate tools is crucial for nurses to perform clinical interventions effectively.” (NM 10).**  “Having high-quality equipment and materials, including new and innovative devices and consumables, is important. In an environment where transparent dressings are used, nurses can more effectively monitor infections and catheter occlusions.” (NM 8)  “Institutions that are more comfortable, where each patient stays in a private room and patient numbers are low.” (NM 8)  **“The patient load and the number of nurses need to be proportional; excessive workload should be avoided.” (NM 7)** |
| Balancing nurses’ professional autonomywith physician-centered care in clinical practice | The restrictive effect of physician-centered workflows on professional autonomy  A bridge facilitating communication between physicians and nurses | The suppressive role of physicians  Autonomy–physician approval conflict in emergency situations  Protecting the scope of responsibility and managing communication  Maintaining boundaries based on mutual respect  Clear delineation of nursing and physician roles  Making nursing care practices visible  Interprofessional collaboration and teamwork | “In the clinical setting, what the physician says is generally followed. Even when I make a suggestion for a patient with fever, I may receive responses such as ‘Are you the one making the decision?” (NM 5)  “In some situations, we have to make rapid decisions regarding the patient because the physician is not present. However, the physician may react by saying, ‘Do not apply restraints without consulting me” (NM 14)  “Although the clinical workflow is generally physician-centered, as the charge nurse I make a deliberate effort to maintain this balance and ensure that care practices remain within nurses’ scope of responsibility.” (NM 9)  “I emphasize within the team that care practices fall under nursing responsibility and that physicians should not intervene in this domain.” (NM 11)  “The charge nurse serves as a bridge facilitating communication between nurses and physicians. When necessary, by clearly defining boundaries, the charge nurse ensures that nurses understand their scope of responsibilities and reminds physicians of these boundaries.” (NM 12)  “Lack of communication in the clinical setting is a significant problem. It is necessary to strengthen nurses’ knowledge base, make nursing care practices more visible, and provide training for physicians to raise awareness of nurses’ roles in clinical decision-making.” (NM 13)  “Recognizing their distinct professional roles, nurses and physicians should work collaboratively for the patient’s benefit. As nurses have the most continuous patient contact, shared interprofessional collaboration enhances both autonomous practice and patient safety. (NM 10) |
